# Long‐term stability and characteristics of behavioral, biochemical, and molecular markers of three different rodent models for depression

**DOI:** 10.1002/brb3.1508

**Published:** 2019-12-22

**Authors:** Han Zhu, Yanlin Tao, Tingting Wang, Jin Zhou, Yingwen Yang, Lin Cheng, Huirong Zhu, Weiqi Zhang, Fei Huang, Xiaojun Wu

**Affiliations:** ^1^ Shanghai Key Laboratory of Compound Chinese Medicines the Ministry of Education (MOE) Key Laboratory for Standardization of Chinese Medicines Shanghai R&D Center for Standardization of Chinese Medicines Institute of Chinese Materia Medica Shanghai University of Traditional Chinese Medicine Shanghai China; ^2^ Center for Counseling and Development Department of Student Affairs Shanghai University of Traditional Chinese Medicine Shanghai China; ^3^ Department of Psychiatry Laboratory of Molecular Psychiatry University of Münster Münster Germany

**Keywords:** 5‐HT, corticosterone, depression model, glucocorticoid, olfactory bulbectomy, UCMS

## Abstract

**Objective:**

The present study was designed to explore the long‐term differences between three mouse models for depression.

**Method:**

In the present study, the unpredictable chronic mild stress (UCMS) model, the glucocorticoid/corticosterone model, and the olfactory bulbectomy model were compared at two, three, and five weeks after model induction. Behavioral testing performed included forced‐swimming, tail suspension, open‐field and elevated plus‐maze tests. In addition, 5‐hydroxytryptamine (5‐HT) and dopamine levels, and mRNA and protein expressions related to 5‐HT synthesis, transport, and signaling were analyzed in the hippocampus of tested animals.

**Results:**

Our results revealed that each model demonstrated a specific profile of markers, whereas the stability of them differed over testing time.

**Conclusions:**

Each model provided a unique set of advantages that can be considered depending on the context and aims of each study. Among the three models, the UCMS model was mostly stable and appeared to the best model for testing long‐term depression‐like state.

## Graphical abstract

There were varied degrees of change from behavior to molecular levels in the three models of depressive mice throughout the length of the modeling time. This includes an evaluation of the time period to be investigated when testing new antidepressants.

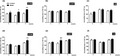

## 1 INTRODUCTION

Depression is a common mental illness that affects more than 300 million people all over the world (Organization, 2018). In the last decades, depression has become a leading cause of disability worldwide and a major contributor to the global disease burden (Organization, 2018). Despite the high prevalence of the disease, its etiology remains unclear; the current treatments are only moderately effective. One reason for the lack of effective treatment is the missing understanding of the underlying neurobiological mechanisms of depression, but also results from the heterogeneity and comorbidity of depression (Krishnan & Nestler, 2008). To improve our understanding, animal models of depression can provide insights into the pathogenic mechanisms of the disease and allow researchers to better tease apart the details of the inner workings of the brain (Nestler & Hyman, 2010).

Based on the limited data regarding the etiology of depression, the available animal models predominantly rely on chronic exposure to stressful experiences such as the unpredictable chronic mild stress (UCMS) model and the high‐dose administration glucocorticoid/corticosterone (CORT) model. In addition, the rodent olfactory bulbectomy (OB) models behavioral and neurochemical changes resembling some of the symptoms observed in depressed patients (Song & Leonard, 2005). Each model has its own set of advantages and disadvantages. In all cases, the depression‐related behavior and neurochemical changes continue throughout the length of the experiment (Mucignat‐Caretta, Bondi, & Caretta, 2006).

The UCMS model includes chronic depression by presenting unpredictable stress stimuli (Gupta et al., 2014) such as multiple, randomly scheduled food and water deprivation, overnight illumination, cage tilting, and other similar stressors that may be associated with human patients (Boyle et al., 2005). In most published studies, the UCMS model induced depression for 2–4 weeks, but some have been tested for up to eight weeks (He et al., 2016; Wu et al., 2017; Xia et al., 2016; Yan et al., 2017; N. Zhang et al., 2018). High levels of serum cortisol, related to elevated hypothalamo‐pituitary‐adrenal (HPA) axis activity, can be associated with depression. The CORT model tests the pharmacologically evoked anxio‐depressive state in animals by application of corticosterone, the rodent functional analogue of cortisol (Krugers, Lucassen, Karst, & Joels, 2010; Rosa et al., 2014) where corticosterone are subcutaneously applied on daily basis for two, three, five, or eight weeks (Gupta, Radhakrishnan, & Kurhe, 2015; Kv et al., 2018; Schloesser et al., 2015; Zhang et al., 2016). The OB models chronic agitated hypo‐serotonergic depression in mice (Lumia, Teicher, Salchli, Ayers, & Possidente, 1992). After bulbectomy, rodent behavioral changes include hyperactivity upon exposure to a novel environment that is sensitive to chronic antidepressant treatment (Song & Leonard, 2005). Analysis of OB rodent models usually occurs after two, four, or twelve weeks after bulbectomy (Nakagawasai et al., 2016; Yurttas, Schmitz, Turgut, Strekalova, & Steinbusch, 2017; Zhou et al., 1998).

Although animals in these models show several phenotypes of depression‐related behavior, including prolonged immobility time in a forced‐swimming test (FST) or tail suspension test (TST), as well as altered neurotransmitter activity and reduced neurotransmitter‐related gene expression, it has not been confirmed whether these characteristics persist for long periods or whether the models show varied qualitative and quantitative similarity to human depression. Comprehensive comparative data are still lacking. In the present study, we analyzed and compared the above three animal models over two, three, and five weeks to investigate the systemic impact on functions ranging from behavior to molecular expression over time. The aim of the study was to gain a better understanding of the pathophysiological processes involved in these animal models. Our results will help researchers choose the most suitable model and determine which time parameters are most useful in each model when evaluating the novel treatment for depression.

## 2 MATERIALS AND METHODS

### 2.1 Animals

In total, 180 male C57BL/6 mice aged five weeks were provided by the Animal Research Center of the Shanghai University of Traditional Chinese Medicine (SHUTCM, Shanghai). All animal experiments were conducted in compliance with a protocol approved by the University Animal Care and Use Committee of Shanghai University of Traditional Chinese Medicine and in accordance with the guidelines of the National Institutes of Health Guide for the Care and Use of Laboratory Animals (National Research Council (U.S.). Committee for the Update of the Guide for the Care and Use ofLaboratory Animals.). They were housed under a 12‐hr dark/ light cycle (light period from 07:00 to 19:00) at room temperature (25 ± 1°C) and provided food and water ad libitum. Unless mentioned otherwise, all mice were randomly housed in groups of four per cage upon arrival for one week to allow habituation until experimental use.

### 2.2 Reagents

Isoflurane was purchased from HeBeiJiuPai Company. Methylprednisolone was obtained from Pfizer (Belgium, NV). Dopamine (DA), and 5‐hydroxytryptamine (5‐HT), 5‐hydroxyindole acetic acid (5‐HIAA), and 3, 4‐dihydroxybenzylamine as internal standard were provided by Sigma‐Aldrich. LC‐MS‐grade acetonitrile and methanol were obtained from Fisher Scientific Co. High‐purity water was provided by a Milli‐Q system. LC‐MS‐grade formic acid was from Tedia Company, Inc.

### 2.3 Establishment of depression models

For each model, mice were weighed weekly. Behavioral testing began two, three, or five weeks after induction of the model as described below.

### 2.4 UCMS model

The modeling method was designed according to literature (Forbes, Stewart, Matthews, & Reid, 1996) with minor modifications. Sixty mice were randomly divided into control (housed four mice per cage) and UCMS group (housed individually). The mice of UCMS group were exposed to a variety of unpredictable mild stressors including the following: body restraint (3 hr), inverted light/dark cycle (48 hr), food deprivation (24 hr), water deprivation (12 hr), electric shock (0.5 A, shock/min, 5 min), damp sawdust (12 hr), tail pinch (1 min), empty cage (no litter in the cage, 24 hr), cage tilting of 45° (24 hr), and soiled cage (24 hr). These stress procedures were randomly scheduled during the experimental period with at least one stressor every day. The mice of control group were housed under normal conditions without specific stimulus.

### 2.5 CORT model

The model was established based on previous glucocorticoid‐induced depression (Kv et al., 2018; Mesripour, Alhimma, & Hajhashemi, 2019) and had been slightly modified. Sixty mice were randomly divided into control and CORT groups. The mice of CORT group mice were intraperitoneally (i.p.) administered 40 mg/kg methylprednisolone consecutively for two, three, and five weeks. The mice of control group were given i.p. saline injections.

### 2.6 OB model

Sixty mice were randomly divided into control and OB groups. The mice in the OB group underwent olfactory bulbectomy while the mice in the control group underwent sham surgery. For bulbectomy, the bilateral olfactory bulbs were removed as described previously (Almeida et al., 2017) with minor modifications. The mice were anaesthetized with isoflurane mixed with oxygen, and an incision made in the overlying skin to expose the skull. Then, a burr hole was drilled on each side, 2 mm from the midline of the frontal bone overlying the olfactory bulbs (2 mm in diameter; 4 mm anterior to bregma). The olfactory bulbs were aspirated using a blunt hypodermic needle and a vacuum pump. Special care was taken to avoid damaging the frontal cortex. Sham‐operated controls underwent all of the same surgical procedures, but the olfactory bulbs were left intact. After the surgery, all incisions were closed with 4–0 vicryl suture. The animals were allowed to recover for two weeks.

### 2.7 Behavioral testing

**Figure 1:**
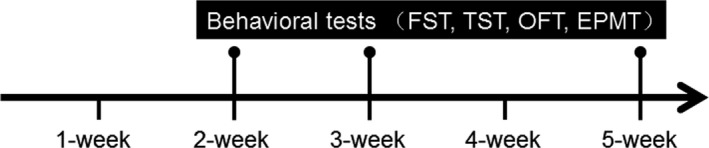
Experimental design for behavioral tests. Abbreviations: EPMT, elevated plus‐maze test; FST, forced‐swimming test; TST, tail suspension test; OFT, open‐field test

Unless mentioned otherwise, all the tests were performed between 09:00 and 12:00. The experimental schedule is shown in Figure 1.

### 2.8 Forced‐swimming test

Mice were individually placed in a glass cylinder (height 30 cm, diameter 20 cm) filled with 20°C water to a height of 20 cm. The animals were allowed to swim for 6 min, and the duration of immobility was quantified from video recordings of the last 4 min. Immobility was defined as floating passively with no active movements.

### 2.9 Tail suspension test

Mice were individually suspended 50 cm above the floor by taping the tail to a horizontal bar for 6 min. The duration of immobility was quantified from video recordings of the last 4 min. Immobility was defined as no active movements except normal respiration.

### 2.10 Open‐field test (OFT)

Mice were individually placed in the center of an open field (50 × 50 × 50 cm). Time spent in the central area and total distance traveled by mice in 5 min were recorded and analyzed by a video‐tracking system (Mobiledatum Inc.). The OFT was used to evaluate anxiety‐related behavior.

### 2.11 Elevated plus‐maze test (EPMT)

The EPMT was performed as described previously (Lister, 1987; Pellow, Chopin, File, & Briley, 1985) to evaluate anxiety‐related behavior. The maze consisted of two open arms (30 × 5 cm), two closed arms (30 × 5 × 15 cm), and a connecting central area (5 × 5 cm), all of which was elevated 60 cm above the floor. Mice were placed in the central area facing an open arm to initiate the test; they were allowed to explore the maze freely for 5 min in a dimly lit room. The number of entries and time spent in open arms were recorded, respectively.

### 2.12 LC‐MS/MS analysis

Twelve hours after the final behavioral tests, the mice were anesthetized with an overdose of 1% pentobarbital sodium. Hippocampi were dissected on ice, and one half of each was processed for LC‐MS/MS analysis. Briefly, the hippocampus was homogenized in a disposable glass tube after adding of 400 μl ice‐cold methanol with 0.1% formic acid and 10 μl internal standard. The homogenate was vortexed for 1 min and then centrifuged at 18,000 × *g* for 10 min at 4°C. The supernatant was transferred and evaporated to dryness under a nitrogen stream. The resultant dry residue was reconstituted in 100 μl of initial mobile phase (0.1% formic acid in water/acetonitrile, 98:2, v/v), and a 10 μl aliquot was injected into the LC‐MS system for analysis under conditions described previously (Huang et al., 2014).

### 2.13 Real‐time PCR analysis

Total RNA was extracted from the second half of the dissected hippocampi of five mice per group using Trizol according to the manufacturer's instructions (Life Technologies). After eliminating trace amounts of DNA contamination with DNase I, the RNA extracts were reverse transcribed into cDNA with a Revert Aid First Strand cDNA Synthesis kit (Fermentas). The synthesized cDNAs were used as templates for real‐time PCR as described previously (Cao et al., 2017). The primer sequences used in PCR are shown in Table 1. Quantitative PCR was conducted with SYBR Premix EX Taq under the conditions suggested by the manufacturer (Life Technologies). The quantity of respective genes was calculated by the comparative Ct method with glyceraldehyde‐3‐phosphate dehydrogenase (GAPDH) used as an internal reference (Ferraz & Fernandez, 2016).

**Table 1:** Primer sequences for real‐time PCR

Genes	Forward primer	Reverse primer
5‐HTR1A	CTCCCTGCTCAACCCAGTTATTTAT	CACTCTTCCTCCACTTCTTCCTTCT
5‐HTR2C	CCTTGCTCCTTCGTTGCTTTCT	ATGTGCATCATTCTGGTCTCCTG
TPH2	TCGAAATCTTCGTGGACTGCG	CGGATTCAGGGTCACAATGGT
SERT	ATGTTGTCCTGGGCGAAGTA	ATGTTGTCCTGGGCGAAGTA
GAPDH	ATGTGTCCGTCGTGGATCTGA	ATGCCTGCTTCACCACCTTCT

Abbreviations: 5‐HTR1A: 5‐hydroxytryptamine receptor 1A; 5‐HTR2C: 5‐hydroxytryptamine receptor 2C; GAPDH: glyceraldehyde‐3‐phosphate dehydrogenase; SERT: serotonin transporter; TPH2: tryptophan 5‐hydroxylase 2.

### 2.14 Western blotting analysis

The second halves of the hippocampi of another five mice per group were homogenized in CelLytic^™^ MT mammalian tissue lysis reagent with protease and phosphatase inhibitors. Thirty micrograms of total protein from each sample were separated by SDS‐PAGE (10% or 12%) and transferred onto PVDF membranes. The membranes were sequentially blocked with 5% bovine serum albumin, incubated with the respective primary antibodies against 5‐HT receptor 1A (5‐HTR1A, Abcam, Cat# ab85615, RRID: AB _10696528, Cambridge, London, UK), 5‐HT receptor 2C (5‐HTR2C, Abcam, Cat#ab137529, RRID: AB_2815031), brain‐derived neurotrophic factor (BDNF, Abcam Cat# ab108319, RRID: AB_10862052), and tropomyosin receptor kinase B (TrkB, Abcam Cat# ab18987, RRID: AB_444716). Thereafter, they were incubated with the appropriate secondary antibodies conjugated with horseradish peroxidase (Life Technologies) for 1 hr at room temperature. The protein bands were visualized with the Immobile Western Chemiluminescent HRP Substrate (Merk Millipore) and quantified with ImageJ 1.46r software (NIH, USA).

### 2.15 Statistical analysis

The data of behavioral tests were processed by Animal behavior analysis system version 2.20 software (Mobiledatum, Shanghai, China). The data are expressed as mean ± standard error of the mean (*SEM*) and analyzed by two‐way analyses of variance or Student's *t* test using GraphPad Prism 6 software. *p* values less than .05 were regarded as statistically significant.

## 3 RESULTS

### 3.1 Body weight gain

**Figure 2:**
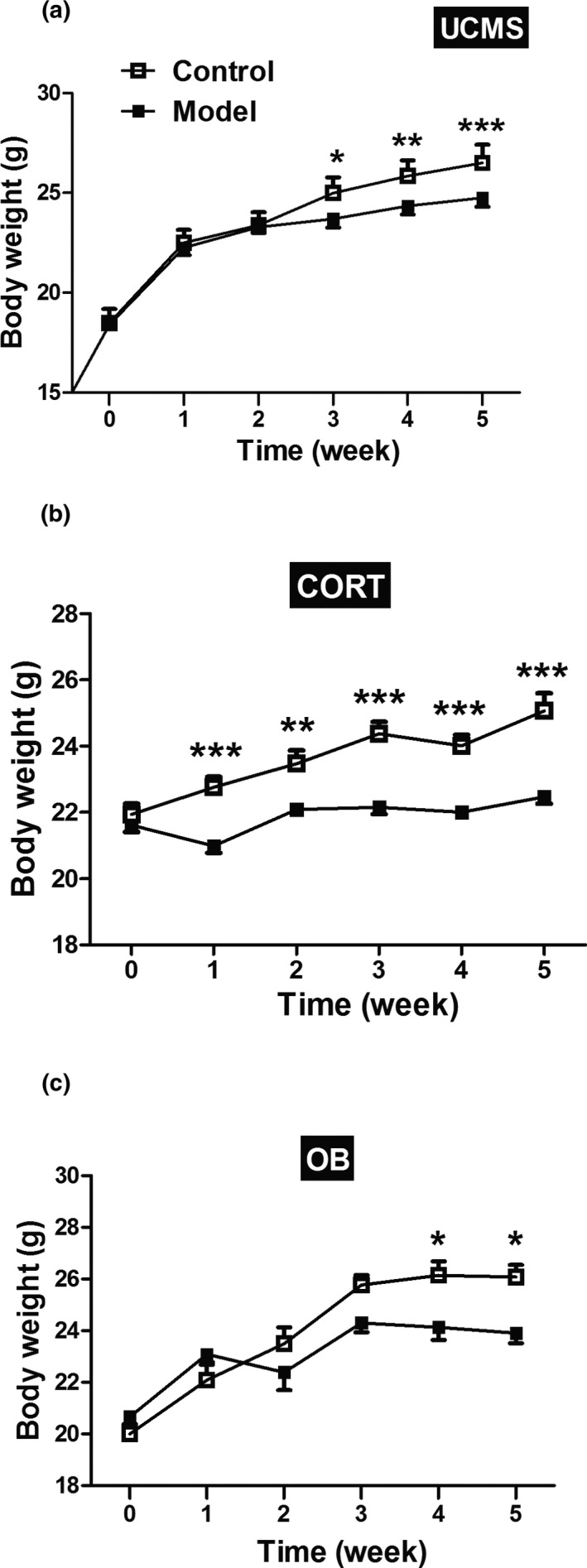
Body weight change curves for the three depression models mice over five weeks. (a) Body weight change of mice in UCMS; (b) Body weight change of mice in CORT; (c) Body weight change of mice in OB. Data are expressed as mean ± *SEM*. *N* = 10 for each group. *, *p* < .05; **, *p* < .01; ***, *p* < .001 versus Control group

As can be seen in Figure 2, all three modeling methods influenced body weight and were affected by modeling time. All model mice exhibited significant weight loss compared to control mice. The UCMS and OB model mice exhibited a decreased body weight gain compared to controls from the third week and the fourth week, respectively (Figure 2a,c, *p* < .05 or *p* < .01 or *p* < .001). In contrast, the CORT model mice demonstrated a decreased weight gain throughout the entire 5‐week administration of methylprednisolone (Figure 2b,* p* < .01 or *p* < .001).

### 3.2 Depression‐like behaviors

**Figure 3:**
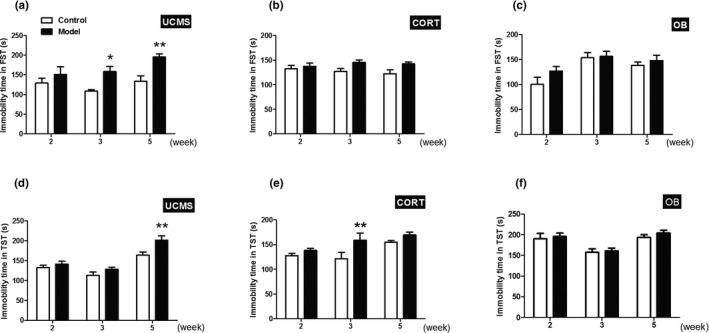
Immobility time in the FST and TST. (a‐c) The immobility time of three model mice with the time course in the FST; (d‐f) The immobility time of three model mice in the TST with time course. Data are expressed as mean ± *SEM*. *N* = 10 for each group and time point. *, *p* < .05; **, *p* < .01 versus Control group

Hopeless behavior of three model mice was determined using FST and TST. The changes of immobility times in FST and TST were different across the three models. For the CORT and OB model mice, the immobility times in the FST were stable and did not differ over time (Figure 3b,c, *p* > .05). However, the immobility times of the UCMS model mice in FST were significantly longer than their controls at week 3 and 5 (Figure 3a, *p* < .05 or *p* < .01). In TST, the immobility time of the OB model mice was similar to their controls (Figure 3f, *p* > .05). In contrast, the UCMS model mice showed a significant increase in immobility time at week 5 when compared with their controls (Figure 3d, *p* < .01), and the CORT model mice displayed a significant increase in immobility time at week 3 when compared with their controls (Figure 3e, *p* < .01).

### 3.3 Anxiety‐like behavior

**Figure 4:**
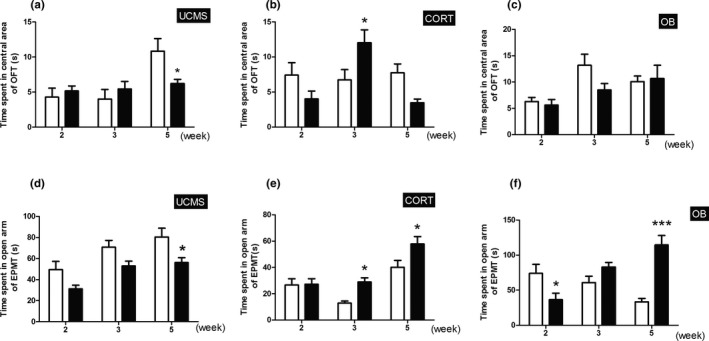
Time spent in central area of the OFT and time spent in open arms of the EMPT. (a‐c) Time spent in the central area of three model mice with time course in the OFT; (d‐f) Time spent in the open arms of the three models with time course in EMPT. Data are expressed as mean ± *SEM*. *N* = 10 for each group and time point. *, *p* < .05; ***, *p* < .001 versus Control group

The time spent in the central area in OFT and the time spent in open arms in EPMT are measures of putative anxiety‐like behavior. In OFT, the UCMS model mice spent less time than their control mice in the central area at week 5 (Figure 4a, *p* < .05). In comparison, the CORT model mice spent more time in the central area than controls at week 3 (Figure 4b, *p* < .05), whereas the OB model mice showed no significant differences over the testing time (Figure 4c, *p* > .05). In EPMT, the UCMS model mice remained in the open arms for a shorter time than their controls at week 5 (Figure 4d, *p* < .05). Both the CORT and OB model mice remained in the open arms significantly longer than their control groups at week 5, as well as at week 3 just in CORT model mice, whereas OB model mice remained in the open arms significantly shorter than their control groups at week 2 (Figure 4e,f, *p* < .05 or *p* < .001).

### 3.4 Locomotor activity

**Figure 5:**
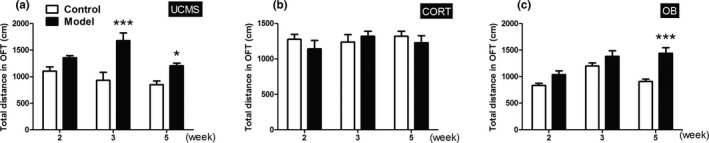
Total distance in the OFT. (a‐c) The total distance of the three model mice with time course in the OFT. Data were expressed as mean ± *SEM*. *N* = 10 for each group and time point. *, *p* < .001 versus Control group

In OFT, the UCMS model mice had significantly longer total traveling distances than controls at week 3 and week 5 (Figure 5a, *p* < .001 or *p* < .05). The OB model mice showed significantly longer traveling distances than control mice at week 5 (Figure 5c, *p* < .001), whereas no significant differences were found between the CORT model mice and their controls (Figure 5b, *p* > .05).

### 3.5 Hippocampal neurotransmitter level

As shown in Table 2, UCMS model samples had significantly lower 5‐HT at week 5 than their controls (*p* < .05), while the 5‐HT level of the CORT model mice decreased at both weeks 3 and 5 (*p* < .05), and the OB model mice had no significant changes in 5‐HT levels in the hippocampus. The ratio of 5‐HIAA/5‐HT (a marker of 5‐HT turnover; Table 3) in UCMS model mice was elevated significantly at week 3 compared to their controls (*p* < .05). The CORT model mice demonstrated a reduced 5‐HIAA/5‐HT ratio at week 3 compared to their controls (*p* < .05). Though the 5‐HT level in the hippocampus of OB model mice did not change compared to controls, the ratio of 5‐HIAA/5‐HT at week 2 was significantly increased in the hippocampus of OB model mice compared to controls, whereas it was significantly reduced at week 5 of modeling while did not change at week 3 (*p* < .05 and *p* < .01, respectively).

**Table 2:** Comparison of hippocampal 5‐HT concentration among the three model groups (ng/g, mean ± S.E.M.)

Time	Group	UCMS	CORT	OB
2‐week	Control	445.226 ± 14.554	180.805 ± 16.104	93.564 ± 3.474
	Model	459.006 ± 16.583	193.409 ± 16.235	104.331 ± 5.422
3‐week	Control	340.366 ± 16.268	298.474 ± 13.537	93.996 ± 5.567
	Model	385.645 ± 13.412	252.636 ± 9.775*	100.343 ± 5.593
5‐week	Control	341.512 ± 15.354	323.637 ± 12.937	74.734 ± 5.875
	Model	285.701 ± 9.441**	307.232 ± 9.422*	82.952 ± 3.092

Note

*N* = 10/group.

* *p* < .05;

** *p* < .01.

**Table 3:** The ratio of 5‐HIAA/5‐HT in mouse hippocampus by model (mean ± *SEM*)

Time	Group	UCMS	CORT	OB
2‐week	Control	0.361 ± 0.023	1.187 ± 0.065	1.257 ± 0.134
	Model	0.319 ± 0.015	1.067 ± 0.046	1.721 ± 0.085*
3‐week	Control	0.374 ± 0.015	1.187 ± 0.065	0.862 ± 0.037
	Model	0.435 ± 0.021*	0.898 ± 0.041*	1.065 ± 0.079
5‐week	Control	0.468 ± 0.020	0.762 ± 0.047	2.018 ± 0.151
	Model	0.525 ± 0.017*	0.712 ± 0.032	1.552 ± 0.040**

Note

*N* = 10/group.

* *p* < .05;

** *p* < .01.

The UCMS model mice showed a significant increase in hippocampal DA levels at week 3 that was significantly reduced at week 5 (Table 4; *p* < .05 and *p* < .01) compared to their controls. The OB model mice displayed reduced hippocampal DA levels at week 3 and increased DA levels at week 5 (*p* < .01) compared to controls. The CORT model mice had significantly lower hippocampal DA at weeks 3 and 5 (*p* < .01) than control mice.

**Table 4:** Comparison of hippocampal DA concentration from the three model mouse groups (μg/g, mean ± S.E.M.)

Time	Group	UCMS	CORT	OB
2‐week	Control	4.595 ± 0.258	6.637 ± 0.573	1.503 ± 0.184
	Model	5.292 ± 0.283	6.467 ± 0.700	1.051 ± 0.168
3‐week	Control	4.098 ± 0.177	5.920 ± 0.392	0.771 ± 0.031
	Model	4.749 ± 0.207*	3.729 ± 0.445**	0.650 ± 0.025**
5‐week	Control	5.396 ± 0.309	3.749 ± 0.283	0.518 ± 0.159
	Model	4.371 ± 0.118**	2.744 ± 0.192* *	0.849 ± 0.125**

Note

*N* = 10/group.

* *p* < .05;

** *p* < .01.

### 3.6 Hippocampal mRNA expression

**Figure 6:**
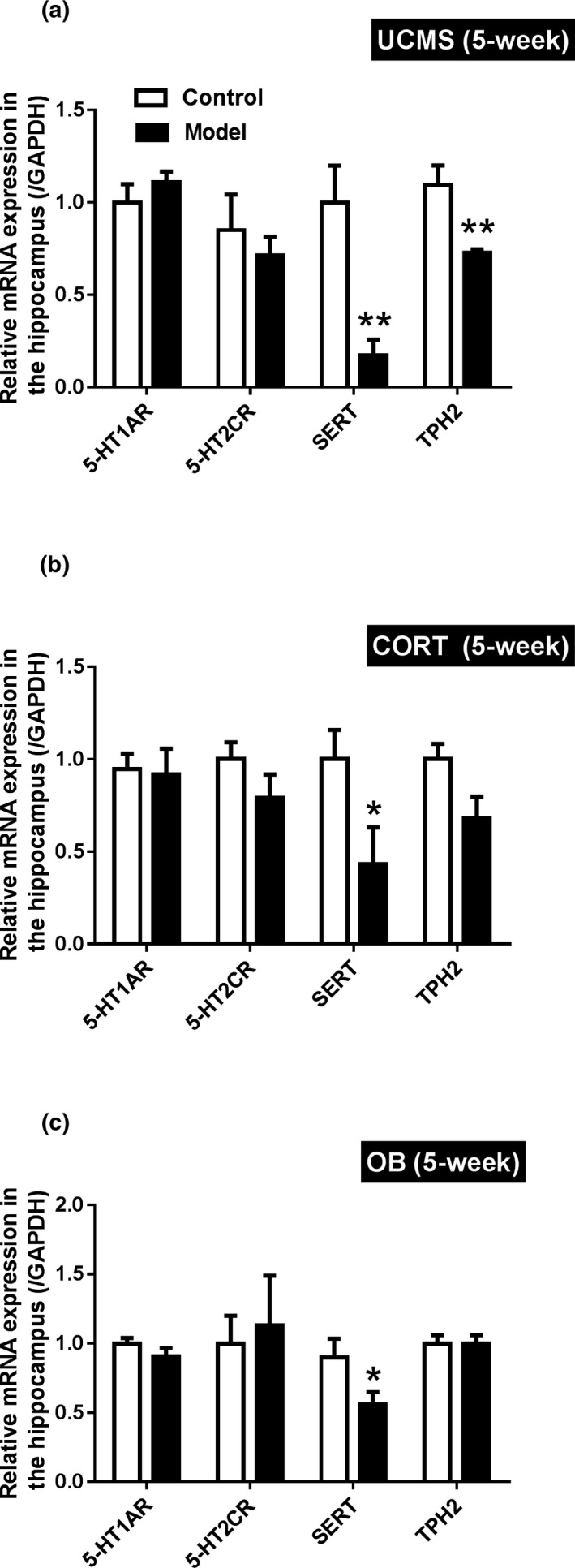
Hippocampal mRNA expressions of 5‐hydroxytryptamine receptor 1A (5‐HTR1A), 5‐hydroxytryptamine receptor 2C (5‐HTR2C), serotonin transporter (SERT) and tryptophan 5‐hydroxylase 2 (TPH2) in the UCMS, CORT and OB model mice. All tissues are from the mice at 5 weeks. Data are expressed as mean ± SEM. *N* = 5 for each group. *, *p* < .05; **, *p* < .01 versus Control group

As shown in Figure 6, mRNA expressions of *5‐HTR1A* and *5‐HTR2C* were unaffected regardless of the model (*p* > .05). However, all three models demonstrated significant reductions in *SERT* (the 5‐HT transporter) expression compared to their controls (*p* < .05 for CORT and OB; *p* < .01 for UCMS). Only the UCMS model mice showed decreased mRNA expression of *TPH2* compared to their controls (*p* < .01), whereas the mRNA expressions of *TPH2* were not changed in hippocampus of CORT and OB mice.

### 3.7 Hippocampal protein expression

**Figure 7:**
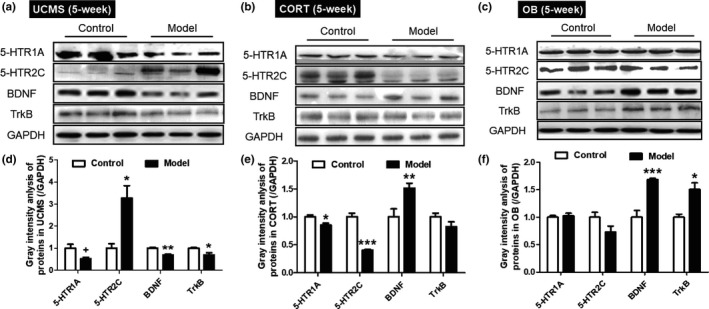
Hippocampal protein expressions of 5‐hydroxytryptamine receptor 1A (5‐HTR1A), 5‐hydroxytryptamine receptor 2C (5‐HTR2C), brain derived neurotrophic factor (BDNF) and tropomyosin receptor kinase B (TrkB) in the UCMS, CORT and OB model mice. All tissues are from the mice at 5 weeks. (a‐c) Western blots of 5‐HTR1A, 5‐HTR2C, BDNF and TrkB in the hippocampus; (d‐f) Gray intensity analysis of the 5‐HTR1A, 5‐HTR2C, BDNF and TrkB in the hippocampus. Data are expressed as mean ± *SEM*. *N* = 5 for each group. ^+^, *p* < .1; *, *p* < .05; **, *p* < .01; ***, *p* < .001 versus Control group

As shown in Figure 7a,d, the UCMS model mice had increased 5‐HTR2C protein expression (*p* < .05) and reduced 5‐HTR1A, BDNF and TrkB expression (*p* < .1, *p* < .01 and *p* < .05, respectively) compared to their controls. The CORT model mice had significant lower 5‐HTR1A and 5‐HTR2C expression (Figure 7b,e, *p* < .05 and *p* < .001), and increased the level of BDNF (*p* < .01) than the controls. The OB model mice showed no differences in 5‐HTR1A and 5‐HTR2C expressions, but displayed significantly higher BDNF and TrkB expression than controls (Figure 7cF, *p* < .001 and *p* < .05).

## 4 DISCUSSION

This study assessed the time courses of different behavioral, biochemical, and molecular parameters in three commonly used rodent depression models. Consistent with most of the literature (Gong et al., 2018; He et al., 2016; Yan et al., 2017), the UCMS model showed depressive‐like behavior and hyperlocomotion from the third week, as well as anxiety‐like behavior at the fifth week. The CORT model demonstrated depressive‐like behaviors and anxiolytic‐like behaviors from the third week, but no differences in locomotor activity in accordance with previous literature (Rosa et al., 2014). Inconsistencies in anxiety‐like behavior repeated elsewhere (Crupi et al., 2011; Wei et al., 2019) may be related to the environmental factors, such as illumination. The OB model mice demonstrated anxiety‐like behavior at the second week, but anxiolytic‐like behavior accompanied by hyperactivity at the fifth week (Zueger et al., 2005). The present data indicated that different behavioral changes emerged at various times after modeling. Notably, there were clear differences between the OB model group and the UCMS/CORT model groups.

Body weight disorder can indicate depression (Blashill & Wilhelm, 2014). In our study, the mice of three models showed decreased body weight gain over time relative to controls; similar as previous literature (Lucca et al., 2008) suggested that decreased body weight gain represents a symptom of depression. The immobility time in the FST and TST are frequently used to represent “behavioral despair” as a marker of depression. The FST is the best‐validated method for assessing depressive symptoms and predicting antidepressant medications’ efficacy (Nestler et al., 2002). We found longer immobility times in the UCMS and CORT models, but not in OB model. Consistent with these results, 5‐HT in the hippocampus of the UCMS and CORT models was significantly lower by week 5, whereas that in the OB model was unchanged over time. One possible reason is that new sensory neurons replaced the old ones, as suggested by receptor cell turnover of 4 ~ 5 weeks (Mucignat‐Caretta et al., 2006).

It has been demonstrated that 85% of patients with depression also experience significant symptoms of anxiety, whereas comorbid depression occurs in up to 90% of patients with anxiety disorders (Gorman, 1996). The OFT and the EMPT are well validated for use in investigating anxiety‐related activity (Belzung & Griebel, 2001; Ramamoorthy, Radhakrishnan, & Borah, 2008; Walf & Frye, 2007). The UCMS model mice showed anxiety‐like behavior at week 5. The total distance moved in both the UCMS and OB models was also higher at week 5, but not in the CORT model. On the contrary, the CORT model mice demonstrated anxiolytic behaviors in both tests, and the OB model mice demonstrated anxiolytic behavior in the EMPT. When rodents are placed in a novel environment, they show feelings of both anxiety and exploration (Belzung & Le Pape, 1994). Our findings suggest the UCMS model mice had increased anxiety, whereas the CORT model mice had decreased anxiety. Interestingly, the OB model mice showed hyperlocomotor activity but less anxious behavior, suggesting reduced defensive behavior in a new environment. However, this is not the only possible interpretation since factors such as locomotor activity may confound anxiety‐like behaviors. Therefore, the extended time spent in the open arms by mice of OB model may be caused by increased overall locomotor activity. 5‐HIAA is the main metabolite of 5‐HT and the ratio of 5‐HIAA/5‐HT represents the metabolic level turnover of 5‐HT. In our experiments, the ratio of 5‐HIAA/5‐HT and DA in the hippocampus of three models exhibited different alterations that lasted throughout the duration of the experiment.

Based on behavioral performance and neurotransmitter changes, we chose to study hippocampal gene and protein expression at week 5. We found reduced *SERT* mRNA expression in all three models, and reduced TPH2 expression only in the UCMS model mice. SERT mediates the clearance and reuptake of 5‐HT during synaptic transmission (S. Ramamoorthy et al., 1993), its reduction in the three models, as showed in the current study, may be related to the impaired release of 5‐HT in these models. As the synthesis of neuronal 5‐HT in the CNS is regulated by TPH2, the decrease of TPH2 may contribute to the decrease of 5‐HT in the UCMS model mice. Among all the 5‐HT receptor subtypes, the 5‐HTR1A and 5‐HTR2C seem to play key roles in depressive neuropathology (Drevets et al., 2000; Hirvonen et al., 2008; Iwamoto, Kakiuchi, Bundo, Ikeda, & Kato, 2004; Martin, Hamon, Lanfumey, & Mongeau, 2014). It has been reviewed that the stimulation of postsynaptic 5‐HTR1A might result in antidepressant‐like effect; but for 5‐HTR2C subtype the data are ambiguous, as both agonists and antagonists induce antidepressant‐like activity (Zmudzka, Salaciak, Sapa, & Pytka, 2018). In the current study, there was no difference in 5‐HTR1A mRNA of all three models, whereas the expression of the 5‐HTR1A proteins in the hippocampi of UCMS and CORT model mice was decreased compared with their control groups. Compared with their control group, the expression of 5‐HTR2C protein increased in UCMS model mice, but significantly decreased in CORT model mice, without changes in 5‐HTR2C on mRNA level. These are partly consistent with the behavioral characteristics of the UCMS and CORT model mice. The above differences reflect the unique profile of 5‐HT level in each model. The UCMS model altered synthesis, transport, and receptor expression, and the CORT model altered transport and receptor functions while the influence of OB in this aspect appeared quite weak.

Previous studies have correlated decreased levels of the neurotrophin BDNF with a higher incidence of depression (Polyakova et al., 2015); in fact, a deficit in BDNF dampens the effect of antidepressants in the UCMS model (Ibarguen‐Vargas et al., 2009). Moreover, the antidepressant agomelatine up‐regulates BDNF gene expression levels in UCMS model rodents (Gumuslu et al., 2014). In current study, significant differences in BDNF were found in all three depression models. Moreover, the BDNF receptor (TrkB) demonstrated significant changes in both UCMS and OB model mice. However, only the UCMS model mice displayed lower levels of BDNF. Decreased TrkB levels may be associated with lower BDNF in these animals and a better simulation by the UCMS model. There are numerous studies demonstrating that hippocampal BDNF decreases after CORT and OB model induction (Gourley, Kiraly, Howell, Olausson, & Taylor, 2008; Lopes et al., 2018). Though the common hypothesis of depression predicts decreased BDNF expression in depression‐related brain areas, our results showed increased BDNF protein in CORT and OB mice at week 5. In addition, some reports indicate that OB modeling in mice leads to increased BDNF levels (Hellweg, Zueger, Fink, Hortnagl, & Gass, 2007). For the CORT model, the decreased BDNF level in the mouse hippocampus could be attributed to the time course of changes in BDNF after chronic stress as reported (Hashikawa et al., 2015).

Although development of convincing, useful animal models for depression represents a major challenge, these three models appear quite useful to further understand disease pathophysiology and for developing treatments based on new molecular targets (Nestler & Hyman, 2010). The UCMS model has high validity but can produce different profiles of behavioral and neuropharmacological effects based on experimental design and end‐point selection (Willner, 2005). In our experiment, the UCMS model was established in single‐caged fed mice stimulated with chronic unpredictable mild stress. According to our experimental results, the UCMS model was more suitable for modeling depression with anxiety and was stable for more than three weeks. The CORT model was more suitable for the study of depression caused by 5‐HT disorders, but it was not stable and might accumulate difficulties after long study duration. The OB model was more potentially better characterized by manic‐depressive symptoms, but it was very unstable at five weeks, which may be related to the unusual plasticity of olfactory system (Mucignat‐Caretta et al., 2006). The OB model's behavioral characterization may suitable, in particular, for testing the presence of depression‐like behaviors in transgenic mice.

To the best of our knowledge, this is the first study comparing the UCMS, the CORT, and the OB models in terms of stability over longer experimental times. Our study emphasized that the awareness of the advantage and disadvantage of the three depression models is essential for ensuring that one's desirable experimental aim and durations can be addressed appropriately.

## CONFLICT OF INTEREST

The authors declare no conflict of interest.

## Data Availability

Data available on request from the authors.
